# Supplementary material for: Plasticity of growth laws tunes resource allocation strategies in bacteria
Source: PLoS Comput Biol. 2024 Jan 8;20(1):e1011735. doi: 10.1371/journal.pcbi.1011735 (PMC10798636; doi:10.1371/journal.pcbi.1011735)
Supplement: S1 Fig — Protein copy number can be converted to protein cost in units of number of amino acids by multiplying copy number with the number of amino acids in each protein. A similar inverse correlation as the one observed for copy number in Fig 1B of the main text, also holds for protein cost. (DOCX) [file pcbi.1011735.s001.docx]

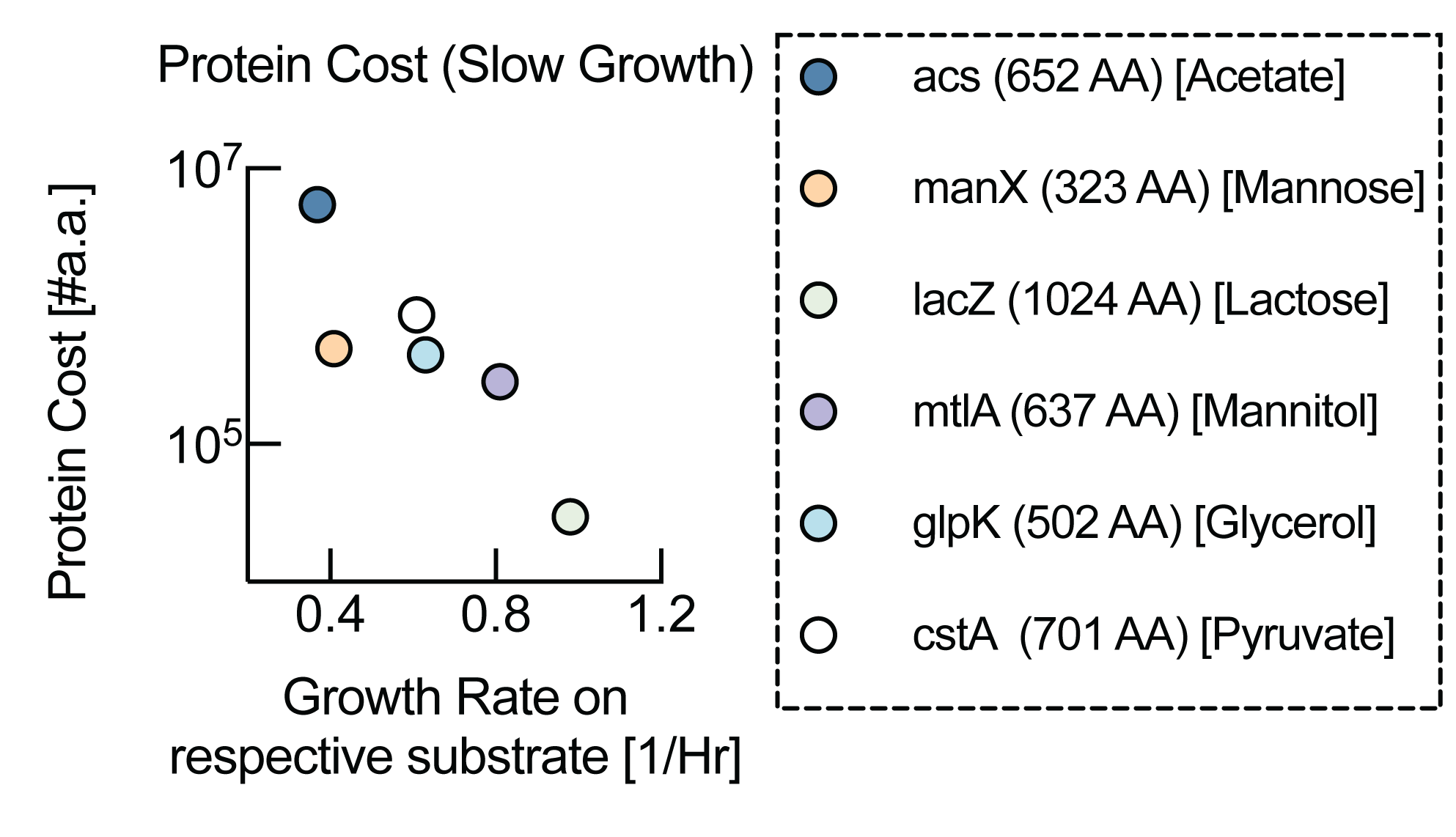


***S1 Fig. Protein cost of substrate-specific transporters and enzymes.*** *Protein copy number can be converted to protein cost in units of number of amino acids by multiplying copy number with the number of amino acids in each protein. A similar inverse correlation as the one observed for copy number in Fig 1b of the main text, also holds for protein cost.*
