# Supplementary material for: Plasticity of growth laws tunes resource allocation strategies in bacteria
Source: PLoS Comput Biol. 2024 Jan 8;20(1):e1011735. doi: 10.1371/journal.pcbi.1011735 (PMC10798636; doi:10.1371/journal.pcbi.1011735)
Supplement: S2 Fig — Growth rates on glucose and mannose minimal medium of different strains. Data points are biological repeats. Unpaired t-test with welch correction was performed. (please see S3 Table for detailed description of strains). (DOCX) [file pcbi.1011735.s002.docx]

*
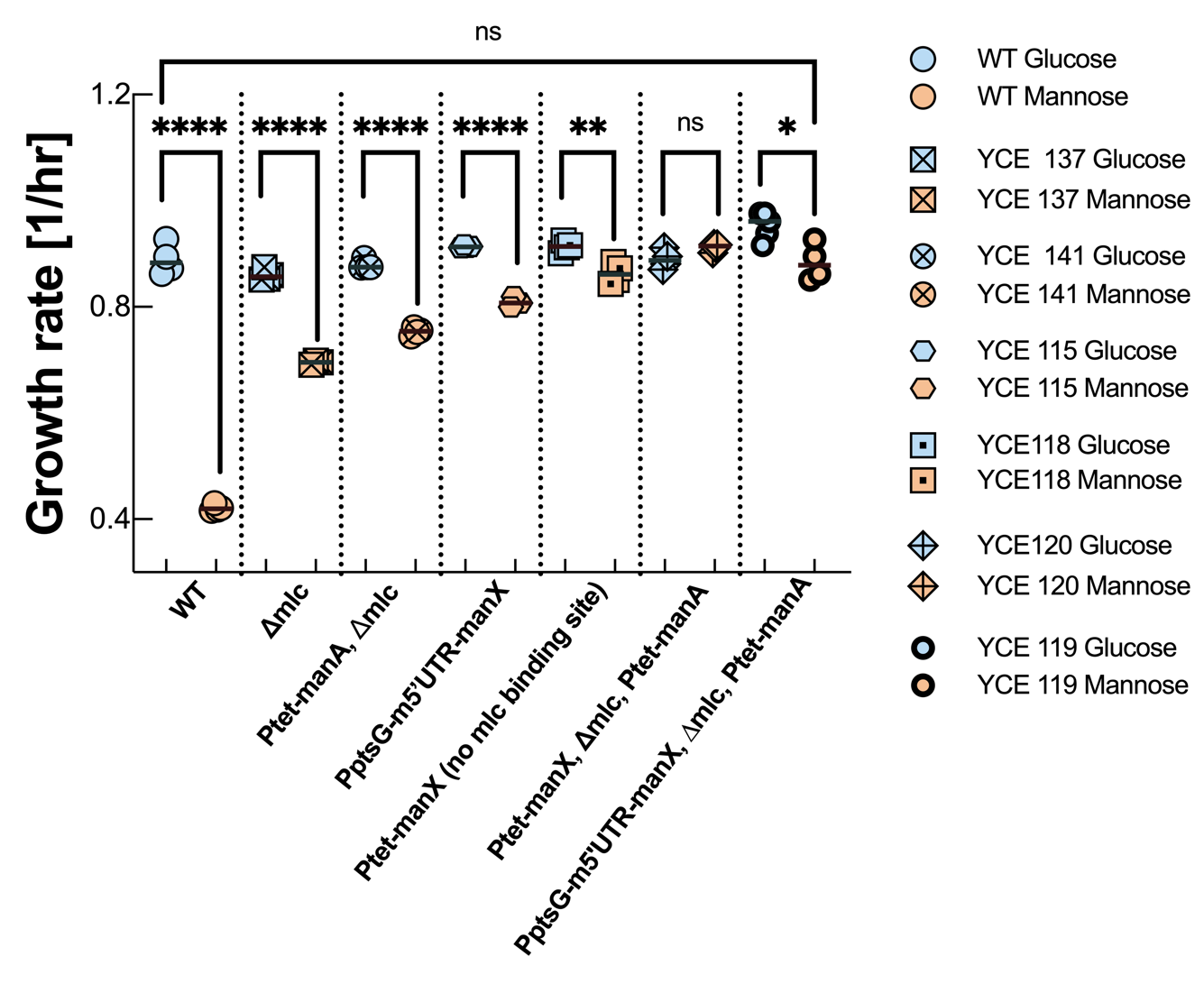
*

**S2 Fig. Growth rate effect of individual genetic modifications and combinations.** Growth rates on glucose and mannose minimal medium of different strains. Data points are biological repeats. Unpaired t-test with welch correction was performed. (please see S3 Table for detailed description of strains).
