# Supplementary material for: Plasticity of growth laws tunes resource allocation strategies in bacteria
Source: PLoS Comput Biol. 2024 Jan 8;20(1):e1011735. doi: 10.1371/journal.pcbi.1011735 (PMC10798636; doi:10.1371/journal.pcbi.1011735)
Supplement: S3 Fig — In Fig 2E of the main text, we plotted endogenous CRP activity measured by Towbin et al. [17] against growth rate on the respective substrate. Endogenous CRP activity and CRP activity where growth rate is maximum are not always the same, as shown by Towbin et al. [17]. Therefore, we plot CRP activity at maximum growth rate against maximum growth rate from Towbin et al. [17] here and find a similar relationship. (DOCX) [file pcbi.1011735.s003.docx]

*
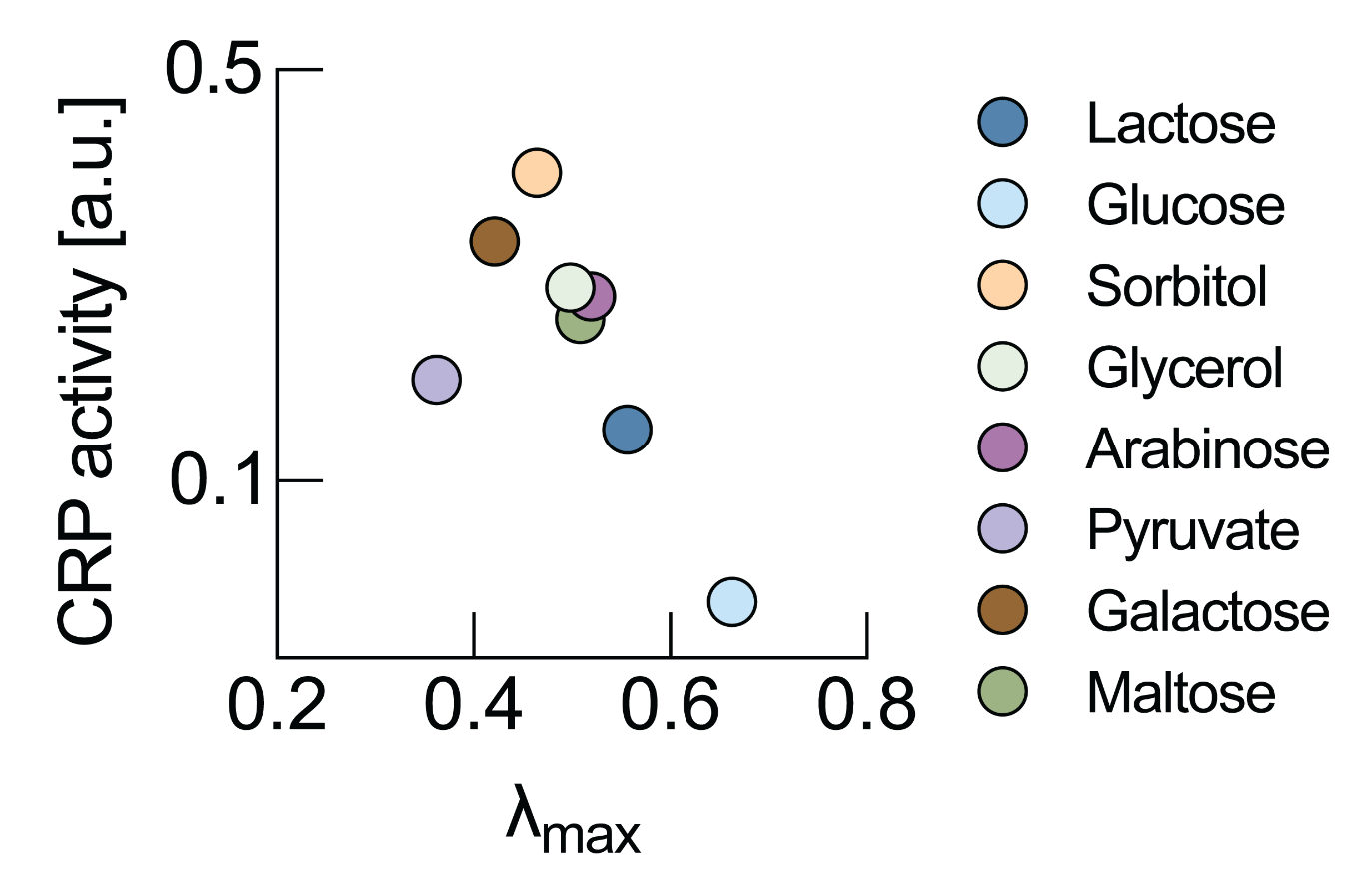
*

**S3 Fig. Crp activity at maxium growth rates for cAMP titrations.** In Fig 2e of the main text, we plotted endogenous CRP activity measured by Towbin et al.[17] against growth rate on the respective substrate. Endogenous CRP activity and CRP activity where growth rate is maximum are not always the same as shown by Towbin et al.[17]. Therefore, we plot CRP activity at maximum growth rate against maximum growth rate from Towbin et al.[17] here and find a similar relationship.
