# Supplementary material for: Plasticity of growth laws tunes resource allocation strategies in bacteria
Source: PLoS Comput Biol. 2024 Jan 8;20(1):e1011735. doi: 10.1371/journal.pcbi.1011735 (PMC10798636; doi:10.1371/journal.pcbi.1011735)
Supplement: S4 Fig — As shown in the main text, many adaptability phenotypes that are lost in the swapped promoter strain (YCE 119) can be rescued by the addition of cAMP (3.5mM) to the growth medium, which upregulates the AD-sector. However, these improvements come at the cost of a reduced growth rate due to the substantial protein cost of the AD-sector. (DOCX) [file pcbi.1011735.s004.docx]

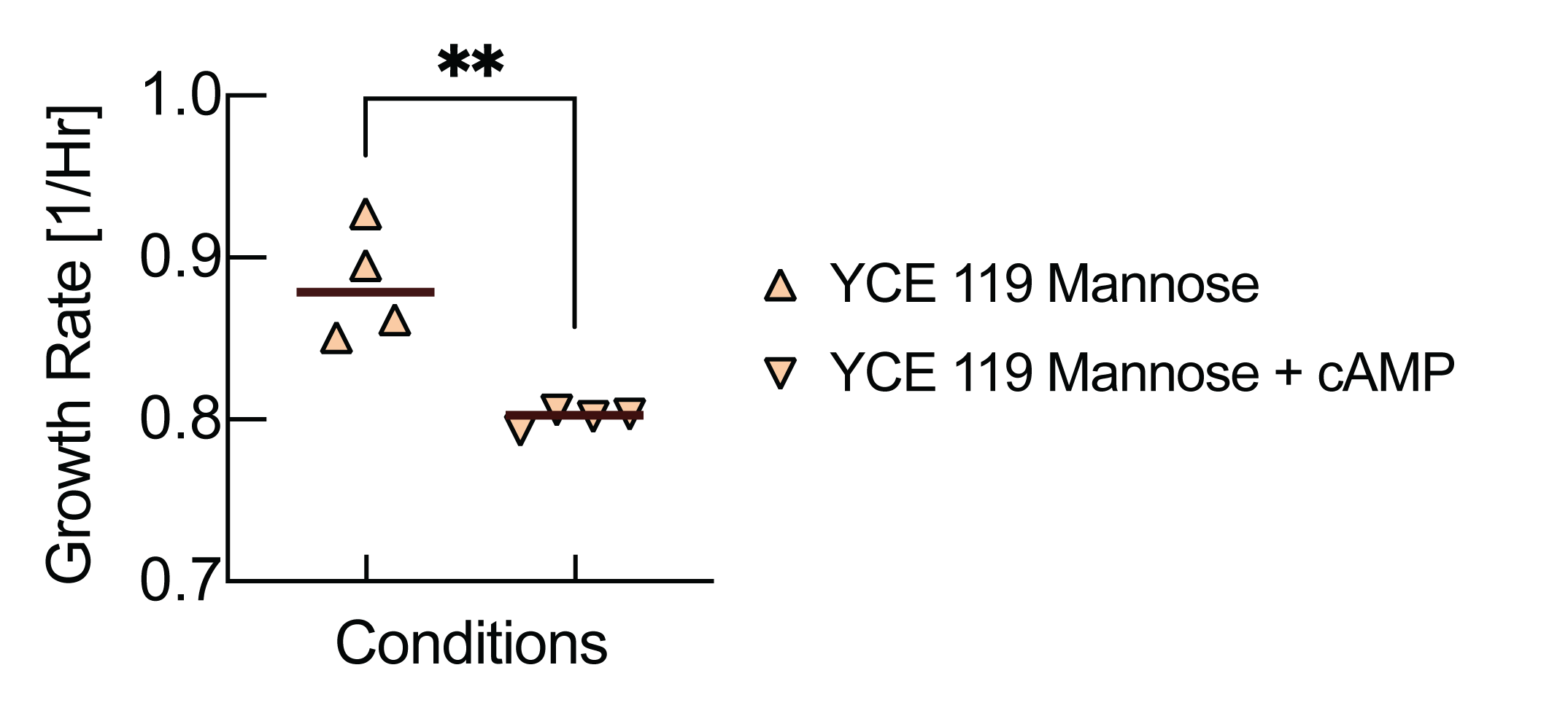


**S4 Fig. Growth rate of swapped promoter strain with the addition of cAMP.** As shown in the main text, many adaptability phenotypes that are lost in the swapped promoter strain (YCE 119) can be rescued by the addition of cAMP (3.5mM) to the growth medium, which upregulates the AD-sector. However, these improvements come at the cost of a reduced growth rate due the substantial protein cost of the AD-sector.
