# Supplementary material for: Plasticity of growth laws tunes resource allocation strategies in bacteria
Source: PLoS Comput Biol. 2024 Jan 8;20(1):e1011735. doi: 10.1371/journal.pcbi.1011735 (PMC10798636; doi:10.1371/journal.pcbi.1011735)
Supplement: S5 Fig — Left panel: Point mutations in glycerol kinase glpK result in faster growth rates on glycerol [25–27]. Resulting growth rates are effectively identical to growth rates of the wildtype on glucose. Data was replotted from Basan et al. [7]. Note that these experiments were performed in MOPS buffered medium, that results in somewhat faster growth rates in all conditions. Right panel: Knockout of the regulator of gluconeogenesis Cra results in faster growth rates on glucose and mannose. Growth rate of the Cra knockout strain on glucose is effectively identical to the growth rate of the wildtype on glucose. Data was replotted from Basan et al. [22]. (DOCX) [file pcbi.1011735.s005.docx]

# *
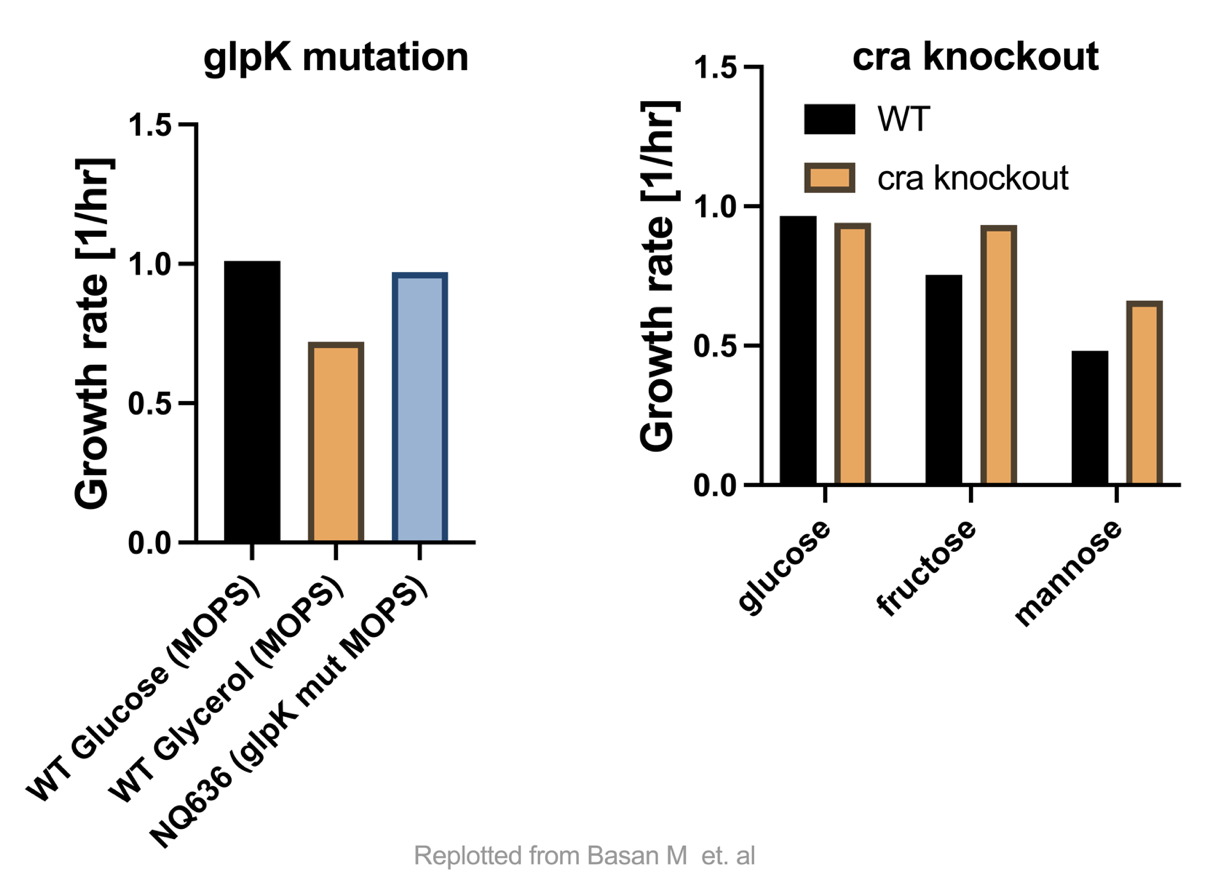
*

**S5 Fig. Growth rate improvements on other substrates.** **Left panel:** Point mutations in glycerol kinase glpK result in faster growth rates on glycerol[25–27]. Resulting growth rates are effectively identical to growth rates of the wildtype on glucose. Data was replotted from Basan et al.[7]. Note that these experiments were performed in MOPS buffered medium that results in somewhat faster growth rates in all conditions. **Right panel:** Knockout of the regulator of gluconeogenesis Cra results in faster growth rates on glucose and mannose. Growth rate of the Cra knockout strain on glucose is effectively identical to the growth rate of the wildtype on glucose. Data was replotted from Basan et al. [22].
