# Supplementary material for: Plasticity of growth laws tunes resource allocation strategies in bacteria
Source: PLoS Comput Biol. 2024 Jan 8;20(1):e1011735. doi: 10.1371/journal.pcbi.1011735 (PMC10798636; doi:10.1371/journal.pcbi.1011735)
Supplement: S1 Table — Protein copy number calculation of the main carbon transporting enzyme (or the first enzyme in the primary carbon degradation pathway) from Li at. al. [12]. (DOCX) [file pcbi.1011735.s006.docx]

| **S1 Table** | | | | | |
| --- | --- | --- | --- | --- | --- |
|  | **Copy numbers in MOPS minimal media (from Li et. al.)]1]** | **Number of amino acids** | **Calculated fold change at 0.45 growth rate (from Hui et. al.)[2]** | **Copy number**  **(at growth rate 0.45)** | **Protein cost**  **(at growth rate 0.45)** |
| **Calculation key🡪** | **X** | **Y** | **Z** | **X • Z** | **(X • Z) • Y** |
| **Gene** |  | | | | |
| **acs** | 643 | 652 | 13.10 | **8423.06** | **5491832.64** |
| **manX** | 2117 | 323 | 0.72 | **1527.21** | **493287.88** |
| **lacZ** | 10 | 1024 | 2.90 | **28.98** | **29679.64** |
| **mtlA** | 197 | 637 | 2.25 | **442.90** | **282129.51** |
| **glpK** | 129 | 502 | 6.84 | **881.85** | **442688.25** |
| **cstA** | 177 | 701 | 6.97 | **1233.16** | **864444.46** |

. **S1 Table. Protein copy number calculation.** Protein copy number calculation of the main carbon transporting enzyme (or the first enzyme in the primary carbon degradation pathway) from Li at. al.[12].
