# Supplementary material for: Plasticity of growth laws tunes resource allocation strategies in bacteria
Source: PLoS Comput Biol. 2024 Jan 8;20(1):e1011735. doi: 10.1371/journal.pcbi.1011735 (PMC10798636; doi:10.1371/journal.pcbi.1011735)
Supplement: S2 Table — Fold change calculation of the main carbon transporting enzyme (or the first enzyme in the primary carbon degradation pathway) at slow growth rate (0.45) from Hui. et. al. [6]. (DOCX) [file pcbi.1011735.s007.docx]

| **S2 Table** | | | |
| --- | --- | --- | --- |
|  | **Fold change calculation from Hui et. al.** | | |
|  | **0.45** | **1.04** | **🡨Growth rate** |
| **Calculation key🡪** | **a** | **e** | **🡨Expression level** |
|  | **a/e** | **e/e** | **🡨Fold change** |
| **Gene** |  |  |  |
| **acs** | 9.98 | 0.76 | 🡨expression level |
|  | **13.10** | 1 | 🡨Calculated fold change |
| **manX** | 0.78 | 1.08 | 🡨expression level |
|  | **0.72** | 1 | 🡨Calculated fold change |
| **lacZ** | 2.44 | 0.84 | 🡨expression level |
|  | **2.90** | 1 | 🡨Calculated fold change |
| **mtlA** | 2.22 | 0.99 | 🡨expression level |
|  | **2.25** | 1 | 🡨Calculated fold change |
| **glpK** | 4.95 | 0.72 | 🡨expression level |
|  | **6.84** | 1 | 🡨Calculated fold change |
| **cstA** | 6.23 | 0.89 | 🡨expression level |
|  | **6.97** | 1 | 🡨Calculated fold change |

***S2 Table. Protein copy number fold changes across growth rates.*** *Fold change calculation of the main carbon transporting enzyme (or the first enzyme in the primary carbon degradation pathway) at slow growth rate (0.45) from Hui. et. al.* [1]*.*

Reference

1. Hui S, Silverman JM, Chen SS, Erickson DW, Basan M, Wang J, et al. Quantitative proteomic analysis reveals a simple strategy of global resource allocation in bacteria. Mol Syst Biol. 2015;11. doi:10.15252/msb.20145697)
