# Supplementary material for: Plasticity of growth laws tunes resource allocation strategies in bacteria
Source: PLoS Comput Biol. 2024 Jan 8;20(1):e1011735. doi: 10.1371/journal.pcbi.1011735 (PMC10798636; doi:10.1371/journal.pcbi.1011735)
Supplement: S3 Table — (DOCX) [file pcbi.1011735.s008.docx]

| S3 Table |  |  |  |  |
| --- | --- | --- | --- | --- |
| Strain | Promoter | 5’ UTR | Expressed gene | Deletion |
| YCE115 | PptsG | m5'UTR | manX | - |
| YCE118 | Ptet | - | manX | - |
| YCE119 | PptsG | m5'UTR | manX | mlc |
|  | Ptet | - | manA |  |
| YCE120 | Ptet | - | manX | mlc |
|  | Ptet | - | manA |  |
| YCE137 | - | - | - | mlc |
| YCE141 | Ptet | - | manA | mlc |

***S3 Table. Detailed description of the strains used in S2 Fig.***
