# Supplementary material for: Plasticity of growth laws tunes resource allocation strategies in bacteria
Source: PLoS Comput Biol. 2024 Jan 8;20(1):e1011735. doi: 10.1371/journal.pcbi.1011735 (PMC10798636; doi:10.1371/journal.pcbi.1011735)
Supplement: Attachment — Submitted filename: Response to reviewers.docx [file pcbi.1011735.s009.docx]

*Reviewer #1: In the paper "Plasticity of growth laws tunes resource allocation strategies in bacteria" the authors show that differences in growth of bacteria between nutrient sources of different "quality" are not a result of the nutrient's inherent metabolic property but likely a result of sub-optimal gene expression resource partitioning. They demonstrate this in particular for mannose as a carbon source. Mannose being a hexose that converts to fructose-6-phosphate thus entering glycolysis, their ultimate conclusion is fairly intuitive, but it is good to have it demonstrated. I have the following comments on the work.

1. The authors should interpret their findings in light of this classic work from Fred Blattner's lab, published nearly 20 years ago.* [*https://www.jbc.org/article/S0021-9258(20)69269-9/fulltext*](https://www.jbc.org/article/S0021-9258(20)69269-9/fulltext)*. This work shows how the transcriptional program of E. coli progressively activates more genes as the 'quality' of carbon source declines. It also shows how RNAP clustering at the highly expressed rRNA genes also declines as the c-source quality decreases. Both these findings are intimately related to the present work. In addition, Blattner's team's work has also used a diverse set of carbon sources.*

This is a great suggestion. Thank you for pointing out this reference. We agree that it should be cited in this work, and we have included it in our revised manuscript and discuss it in detail.

*2. I feel that the general conclusion that the authors have come up with will need more support from different categories of carbon sources, similar to the above-cited work from Blattner's laboratory. If not, it should be clearly stated in the abstract and introduction that their experimental proof applies to hexoses and not broadly among different substrates / nutrients - this itself is valuable.*

In our revised manuscript, we have done both. We have added growth rates from several other genetic perturbations that enhance growth rates on other substrates, including non-hexose, non-PTS substrates (Fig. S5). For example, for growth on glycerol, single point mutations in glycerol kinase GlpK result in growth rates on glycerol identical to those of glucose. This observation has long been puzzling, but we show here that it is not an exception. Another example is the Cra knockout growing on fructose that also results in growth rates identical to those on glucose.

At the same time, we agree with the reviewer that it is impossible to provide a comprehensive test of all substrates of *E. coli* to confirm our hypothesis in full generality. Testing a single substrate is a substantial experimental effort requiring chromosomal replacement of all the promoters in the substrate pathways (see Fig. S2). We do want to point out that these are highly non-trivial experiments and there is no guarantee of success that there will be indeed enhanced growth rates. Not finding enhanced growth rates is not conclusive because the expression levels may not be right or there could be toxic side-effects or other regulatory elements interfering with growth rate.

We have therefore carefully reformulated the abstract, introduction and discussion, to make it clear that our hypothesis appears to hold at least for a subset of substrates, but we cannot rule out that substrates exist that are truly intrinsically ‘energy’-poor or difficult to catabolize.

Nevertheless, we are doubtful that this is the case. The likely candidates for such intrinsically ‘poor’ substrates are gluconeogenic TCA cycle substrates that require a complete rerouting of central metabolism rather than the expression of a few enzymes in a small, dedicated pathway. However, as we quantified in one of our previous works [Schink et al., (2022). Glycolysis/gluconeogenesis specialization in microbes is driven by biochemical constraints of flux sensing. Molecular systems biology], other bacteria like *Pseudomonas aeruginosa* readily grow on these substrates at similar growth rates as *E. coli* growth rate on glucose. Conversely, these bacteria grow quite slowly or not at all on supposedly ‘rich’ glycolytic substrates, including glucose, consistent with our evolutionary/ecological interpretation of nutrient quality.

*3. The authors discuss protein cost purely from its expression levels. However, I understand that the bacteria were grown in minimal media without external amino acids. In this case, there should also be cost arising from the biosynthesis of amino acids. This, to the best of my understanding, has not been incorporated.*

Thank you. We realized we did not explain this point in the submitted version of the manuscript.

Biosynthesis of amino acids and other parts of the protein synthesis pathway can the thought to be included in the R-sector because their protein cost scales the same way as ribosomes.

We have now clarified this in our revised manuscript.

*4. The authors activate mannose metabolism using three different mutations, one of which - introducing a ter promoter- appears to be not the most relevant to sugar metabolism. It appears that Mlc, which the authors have deleted, regulates some PTS components involved in mannose metabolism as well. These make their results difficult to interpret. What are the effects of the individual mutations?*

Originally, our goal was to achieve the maximum growth rate improvement, so tried to simultaneously improve all possible bottlenecks.

We have now added the growth rates of the individual genetic modifications and some combinations in our revised manuscript (Fig. S2). Interestingly, Mlc binding sites repress expression of enzymes for mannose utilization. This is in our opinion a clear indication that sub-maximum growth on mannose is an ‘intentional’ strategy, shaped by evolutionary pressure, rather than representing than an accident or insufficient adaptation to mannose as a substrate.

The same could be said for the FBP binding site in GlpK that reduces growth rates on glycerol.

*5. An alternative approach would be to perform laboratory evolution of the bacterium in mannose as the sole carbon source. I am pretty sure it would find mutations that make it grow better. This in itself would indicate that slow growth in mannose is not because of mannose being mannose. Characterisation of individual mutations arising and their combinations thereof would be a powerful approach towards characterising various strategies that enable the bacterium to deal with the bottlenecks that stultify growth in mannose.*

Yes, we agree that this would be an alternative approach. We may pursue such experiments in a follow-up study. These experiments are more technically challenging than it seems because bacteria need to be prevented from entering stationary phase, which selects for survival rather than the fastest growth rate.

Our colleague Johan Paulsson has built a turbidostat for long-term evolution and is currently conducting similar experiments in fast growth conditions. We want to apply this setup to evolution on poor substrates in the future.

*Reviewer #2: In this contribution the authors explore the notion of "nutrient quality", as it appears in resource allocation models, based on experimental results and analysis of data from the literature. The main question is to assess whether "nutrient quality" is determined by fundamental biochemical limitations or rather by a genetic program shaped by evolution and potentially adaptable to new environments. By analyzing the response of a mutant with upregulated growth on a poor carbon source, and its adaptability phenotypes, they conclude that "nutrient quality" is not an intrinsic biochemical property but rather the result of evolution.

I find the study interesting, but with main limitations as follows:

- The three-compartment resource allocation model that authors start from has been established based on a linear constraint among the size of the three compartments that manifests itself from experimental evidence. In the authors contribution, this model is refined into a multiple compartment model which by nature also corresponds to introducing linear relationships among the compartments. However, the authors' arguments seem to me mostly qualitative, not quantitative. Thus the refined model seems a bit vague to me: How does the size of the many compartments change in different conditions, and how do these changes relate with growth rate?
This concern also relates with the next.*

*- For the targeted venue, what is the computational element (or contribution) of the paper? It seems to me that, besides standard data analysis, the computational nature is limited to the review of an existing growth rate /resource allocation model that is later discussed and refined purely based on qualitative considerations*

It is true that we are not introducing the growth law model *de novo* in this work and are simply proposing a refinement. However, we are adding completely new experimental results that shed new light on the mechanistic underpinnings and the meaning of nutrient quality.

The original growth law paper by Scott et al. [Scott M, Gunderson CW, Mateescu EM, Zhang Z, Hwa T. Interdependence of cell growth and gene expression: Origins and consequences. Science (1979). 2010;330: 1099–1102. doi:10.1126/science.1192588] is often considered to be a seminal work with over 1300 citation. Growth laws also remain a central theme at conferences and meetings related to bacteria and in particular, mathematical models for various aspects of microbial growth. Hence, a better understanding of the concept of nutrient quality, which is at the heart of the growth laws is arguably of broad interest to theorists and the modeling community.

Moreover, we think there is substantial confusion around this concept currently existing the scientific community that must be rectified. This is supported by the observation that we see papers submitted and published, with models that are *de facto* ruled out by the new data provided in this work. To provide just one examples, models involving limited by membrane capacity or any model that assumes growth rate maximization as a central premise.

There undoubtedly widely held misconceptions about what limits growth rates of microbes among both experimentalists and theoreticians. We therefore think our work offers substantial added value by narrowing down classes of possible models to understand growth rate.

*- Authors base their experimental evidence on the construction of a mutant with faster growth on mannose, which amounts to replacing the chromosomal promoter driving a mannose PTS permease with the promoter of the glucose PTS transporter PptsG. This very circumscribed genetic modification seems to me in contrast with the authors' statement at the end of the introduction that "[changing] growth rates by simply changing the expression level of carbon-specific transporters and metabolic enzymes [is] challenging [...] because metabolic pathways of individual substrates typically involve many enzymes and transporter that need to be expressed in concert". Turned another way, how robust is the experimental analysis to the fact that changes in nutrient quality cannot be confined to changes in a single element of the corresponding metabolic chain?*

Improving the growth rate on any substrate is not a simple feat. We needed to upregulate the entire pathway for mannose utilization, including the ManX, ManY, ManZ operon and ManA operon and remove the disruptive effect of Mlc. All of these modifications needed to be done at a chromosomal level because plasmids themselves entail a replication cost that slows down growth rates, making the results impossible to interpret. For these reasons, showing growth rate improvement even for one carbon source is a substantial experimental effort with no guarantee of success at the outset. A negative result does not mean that the growth rate cannot be improved, making this a difficult and risky experimental project.

In our revised manuscript, we have added additional examples of improved growth rate on other substrates that were discovered by accident in the past (Fig. S5). We have also added a growth rates resulting from individual genetic modifications and certain combinations (Fig. S2).

It is impossible to provide an exhaustive list of substrates on which growth rates can be improved, but these data demonstrate that our hypothesis is correct at least for a subset of substrates of *E. coli*. We cannot rule out the existence of substrates that are truly energy poor or very expensive to metabolize. We now explicitly point this out in our revised manuscript.

*- I find the term "nutrient quality" confusing for the discussion that the authors are making. For instance, "nutritional capacity" (as per your reference [4]) seems better adapted to me, since this expression is not solely associated with the nutrient itself (as in "nutrient quality") but rather with the nutrient and the nutritional efficiency resulting from the corresponding metabolism.*

Nutrient quality is the term more frequently used by microbiologists, so we prefer to frame the question around this term. What makes a substrate a ‘rich’ substrate and what makes it a ‘poor’ substrate? We think that the word ‘capacity’ makes things less clear, in fact misleading because it implies that there is some limited capacity of the bacterium to metabolize this substrate. The point of our paper is precisely that there is not.

If the reviewer insists, we agree to change the wording.

*Also I wonder why the authors do not explicitly discuss the chemical composition of substrates as a possible defining part of their "nutritional capacity"; Is this aspect implicitly captured by the hypothesis that "Nutrient quality could be an intrinsic carbon-specific property such as proteome cost of the catabolic pathways, required for metabolizing the substrate and therefore determined by
fundamental enzymatic properties like the maximum catalytic rate V max of these metabolic enzymes and transporters."? This hypothesis seems more related with the cost of metabolizing the substrate rather than with the intrinsic substrate richness*

Chemical composition, energy content, cost of catabolism are typically used to explain different growth rates on different substrates and this is why we discuss them in our manuscript.

However, the central message of our work is that this explanation can be ruled out, at least for a subset of substrates and possibly for all substrates. Explanations like chemical composition, energy content, enzyme cost of metabolism as the limiting factor of growth rate are inconsistent with the observation that growth rate can be substantially improved. Therefore, none of the above are truly limiting growth rate on these conditions.

*-Partly but not only because of the previous issue, I find the first part of Results unclear. For instance, in the context where this is discussed, it is quite unclear to me why authors say that the rank-ordered anticorrelation between the copy number of carbon transporters and growth rate is a striking finding (¨"Strikingly, when we plotted..."). I find that results and their interpretation starts being clear when starting to discuss results on the swapped promoter strain (from "Next, we measured growth rates of swapped promoter strain..." )*

We agree that this is mysterious.

The rank ordered correlation is exactly what would be expected from protein cost being the limiting factor and a broadly held interpretation of growth laws. However, the promoter swap shows that this is in fact not the case and not really a limiting factor. So these two findings are somewhat confounding.

There appears to be a correlation between core nutrient quality $\kappa_{n}^{*}$ and effective nutrient quality $\kappa_{n}$, but core nutrient quality $\kappa_{n}^{*}$ appears not to be limiting for growth. While these findings are surprising, we think they are important to report.

*- Statement "We propose that different substrates convey information about the safety and reliability of the current growth environment", and related statements: Why "safety and reliability"? I rather understand your arguments in terms of evolution having shaped the genetic program so as to avoid focusing cellular resources on substrates that are on average of little interest, while saving cellular resources to adapt fast to substrates that are on average more profitable*

We are not sure what the reviewer means by ‘substrates of little interest’. Our work shows that *E. coli* can in principle grow equally fast on the slowest substrate mannose as on the fastest substrate glucose.

What makes mannose of little interest? Not the fact that it doesn’t support faster growth. Instead, it appears to be an ‘unreliable’ substrate, by which we mean that it either tends to quickly run out (less profitable?) or is correlated with other forms of stress or danger, warranting a bigger investment in stress response, motility, and the search for alternative substrates. We think that maybe what we refer to as ‘reliable’ is what the reviewer refers to as ‘profitable’.

Based on your suggestion, we have added the term ‘profitable’ to the discussion as it might be clearer to some readers.

*- Fig.3: Wouldn't it be interesting to also report how YCE behaves on glucose? Are the results in such scenario in line with your expectations?*

Yes, this data is found in Fig. 2b. The growth rate is close to the normal growth rate on glucose. It is possibly even a little faster than wildtype growth rate on glucose (see Fig. S2). This is probably due to the knockout of the transcription factor Mlc.  *- I find the title generic and unclear about the point the authors make. I also find the Author summary too similar to the Abstract, it should be more sharply different (less technical) in spirit*

Thank you for pointing this out. We agree and have rewritten the author summary. Please see marked changes in our revised manuscript. We like the current title, but are open to suggestions by the reviewer. *Besides these main issues, I find the paper reasonably well written, except for a few minor typos / lexical inconsistencies scattered in the text that I invite authors to fix.*

*Reviewer #3: I performed this review with the help of a junior group member.

This study by Mukherjee and coworkers uses E. coli experiments and literature data to challenge the concept that fundamental biochemical constraints regulate nutrient quality, the catabolic flux per enzyme mass measured for growth in different defined media. The central finding is that E. coli can achieve comparable growth rates on mannose and glucose when the regulatory system controlling mannose transport is swapped with that of glucose. Consequently, it does not seem appropriate to view mannose as a poorer growth medium.

Instead, the authors conclude that the concept of nutrient quality should be understood as a regulated and ecological property, influenced by the organism's adaptation and preference. In other words, nutrient quality signifies how well a substrate aligns with the metabolic capabilities and regulatory mechanisms of bacteria within a specific ecological context.

I find the study interesting and definitely worth publication on Plos CB, but I have a number of comments that the authors may want to address - listed below.*

Thank you for this assessment and these helpful comments.

*I did not find the order in which things are presented very clear. Most of all it seems important to establish clear distinctions between novel findings and novel hypotheses introduced in this study, and existing findings and hypotheses from prior literature. For example, in Box 1 the distinction between new hypotheses and ingredients taken from the literature is not very clear.*

This is a good point. We have added explicit text boxes and titles throughout the manuscript to make it much clearer which data was plotted and reanalyzed from preexisting datasets.

*Experiments and data analysis from other datasets (mainly Hui et al) form the core of the work. The model, on the other hand, serves mainly as a conceptual framework, as the model itself and its predictions are not really fully formalized, and there is no clear comparison between the model's predictions and the experimental findings. This is ok I think but some of the claims do not appear commensurate with this fact. Adding some clarifications could help the reader.*

It is impossible to comprehensively test of all substrates of *E. coli* to confirm our hypothesis in full generality. We have therefore carefully reformulated the abstract, introduction and discussion, to make it clear that our hypothesis appears to hold at least for a subset of substrates, but we cannot rule out that substrates exist that are truly intrinsically ‘energy’-poor or difficult to catabolize.

Nevertheless, we are doubtful that this is the really the case. The likely candidates for such substrates are gluconeogenic TCA cycle substrates that require a complete rerouting of central metabolism rather than the expression of a few enzymes in a small, dedicated pathway. However, as we quantified in one of our previous works [Schink et al., (2022). Glycolysis/gluconeogenesis specialization in microbes is driven by biochemical constraints of flux sensing. Molecular systems biology], other bacteria like *Pseudomonas aeruginosa* readily grow on these substrates at similar growth rates as *E. coli* growth rate on glucose. Instead, these bacteria grow quite slowly on supposedly ‘rich’ glycolytic substrates, including glucose, consistent with an evolutionary/ecological interpretation of nutrient quality.

*Since no real model testing is performed, it would be seem more accurate to characterize Figures 3 and 4 as consistent with a hypothesis rather than as conclusive evidence towards a model prediction. Protein functional classes that associate to lag, starvation, motility, show trends (Fig 3bc, 4b) in line with the authors' hypothesis, and so do the genetically engineered strains studied here (Fig 3de, 4cd)*

The swapped promoter strain is a direct experimental test of our hypothesis. In addition, we have added other examples where growth rates can be substantially improved (Fig. S5).

Figs. 3&4 demonstrate that growth rates and protein expression changes are reflected in actual adaptability phenotypes. These data suggest a possible reason why growth rates are not maximized. However, it is impossible to definitively prove this hypothesis outside very controlled and technical laboratory evolution experiment, in which objectives other tha n growth rate are selected for.

We have added a cautionary statement on page 21 about Figs 3&4 to clarify this point.

*Adding some formalization may also help. For example manuscript just gives some intuition on what k_n should be given the data. However, it misses a clear definition of "nutrient quality". How should an equation for k_n look like, given the presented results?*

We do think this part is fully formalized in the equations in Box 1c, d, e.

The cell can freely pick the nutrient quality via the core nutrient fraction $f$, which is a parameter that can be freely adjusted. The only constraint is that $f\leq1$.

*More conceptually, the model (if I understood correctly) suggests that the parameter kn, representing nutrient quality, can be adjusted through a specialized sector with minimal associated costs (which can be neglected). Additionally there exists a trade-off between increasing nutrient quality and a separate "adaptability sector" encompassing factors like motility and costs related to lag phases and starvation. A question arises regarding why this trade-off manifests differently for various substrates (glucose vs mannose). Additionally, it raises the question of why the wild-type bacteria can afford such a higher vulnerability by shutting down their adaptability sector in glucose-rich environments.*

We are arguing that nutrient quality directly emerges from this trade-off and the costly adaptability sector. Statistically, environments where the substrate mannose is present, might be such that mannose frequently quickly runs out or where other stresses are likely to emerge. Therefore, the low nutrient quality of mannose is just a reflection of ‘risky’ ecological environments that correlate with mannose, implemented via a regulatory program that tunes on the adaptability sector (see Box 2).

Different substrates reflect different environments and therefore the balance between growth rate and adaptability is shifted. Hence, it is the same trade-off. Growth laws are a direct reflection of this trade-off. Just the balance between growth and adaptability of this trade-off is tuned by the environment and evolution and manifested as nutrient quality.

Note that even on glucose the adaptability sector is not completely shut off. Extrapolating, the growth rate where this occurs for a single carbon source is at around 1.1/hr and we are not aware of any substrates that reach this growth rate.

To summarize, rather than having a different intrinsic quality, the substrates provide the bacterium with information about the environment it finds itself. Nutrient quality is thus a single number that represents everything the bacterium has statistically learned about the safety and reliability of environments that co-occur with this substrate. In other words, the evolutionary pressure from stresses whose occurrence correlates with the occurrence of mannose in natural environments has driven growth rate down on mannose, by rendering it worthwhile to invest more in the adaptability sector.

This idea is illustrated in Box 2.  *Fig 1 and S1 and their interpretation need to be better explained in the text. Currently it appears that some logic steps are missing. First, the fact that the substrates may or may not be present in the medium only appears very late in the text, after all the data have been presented (and it is not said for which data points this is the case, if not all). Second, the possible interpretation in terms of protein cost is dismissed almost before it is introduced and explained clearly. It seems this part would profit from a step-by-step narrative. Also sentences (plots?) detailing the expectations from protein cost are needed.

Specifically, the sentence "However, the observed anticorrelation is still surprising as these substrates were not present in the experimental growth conditions from which protein abundances were determined." is not clear. Do the authors use an extrapolation of the data in order to cover the new substrates? Why one would expect the data to not follow the growth law for the P sector? Can the authors show the comparison between their data in figure 1B and the growth law? Moreover, are the authors talking about the P sector as a whole or about the specific transporters and pathways of each considered substrate?*

We have reworked the discussion of this point and hope it is now clearer in our revised manuscript. We agree that this is a confounding observation. However, this is precisely why we think it is important to report.

Fig. 1b shows the absolute expression level of transporters and enzymes in the same slow growth condition when these substrates are not present. Remarkably, this abundance anti-correlates with growth rate on these respective substrates, reflecting their nutrient quality.

The result is new and is not obvious. It suggests that transporters and enzymes of poor substrates have a higher expression level, in general, even when those substrates are not present.

While not predicted by the growth laws, this interpretation is consistent with the idea that nutrient quality is rooted in enzyme cost (i.e. inefficient enzymes require higher abundances).

However, as we show later in the manuscript, enzyme cost does not appear to be limiting growth rates.

Perhaps if it is advantageous for bacteria to grow slowly on certain substrates, there is less evolutionary pressure for optimizing metabolic enzymes of these pathways.

*The explanation of the genetic system is also not very clear, and lacks a clear question statement e.g. "we ask whether growth rates of E. coli on mannose were limited by the cost of producing the necessary proteins for substrate transport and metabolism, or if they were limited by the available capacity for substrate transport". The PTS system is not defined.*

Yes, these are excellent suggestions. We have added them in our revised manuscript.  *Regarding Figure 3, it would be interesting to see if the relation between growth and death rate found in Biselli et al still holds for the "swapped" strain (the starvation procedure appears to be the same as in Biselli et al.)*

It will probably hold, since the growth rates and death rates are precisely swapped.  *Minor Remarks


In the paragraph after Figure 2, there is a bit of confusion regarding the sectors P, C and C**

Thank you. We have checked this paragraph and were not able to find the problem. Please let us know if this is not resolved.
